# Decoding thermal properties in polymer-inorganic heat dissipators: a data-driven approach using pyrolysis mass spectrometry

**DOI:** 10.1080/14686996.2024.2362125

**Published:** 2024-06-13

**Authors:** Yusuke Hibi, Yasuhiro Tsuyuki, Satoshi Ishii, Eiichi Ide, Masanobu Naito

**Affiliations:** a Data-driven Polymer Design Group, Research Center for Macromolecules and Biomaterials, National Institute for Materials Science (NIMS) Tsukuba, Ibaraki, Japan; b Green Processing Research Department, Production Engineering MONOZUKURI Innovation Center, Research and Development Group, Hitachi, Ibaraki, Japan

**Keywords:** Composite material, polymer, heat conductivity, pyrolysis mass spectrometry

## Abstract

Polymeric materials can boost their performances by strategically incorporating inorganic substances. Heat dissipators are a representative class of such composite materials, where inorganic fillers and matrix polymers contribute to high thermal conductivity and strong adhesion, respectively, resulting in excellent heat dissipation performance. However, due to the complex interaction between fillers and polymers, even slight differences in structural parameters, e.g. dispersion/aggregation degree of fillers and crosslink density of polymers, may significantly impact material performance, complicating the quality management and guidelines for material developments. Therefore, we introduce pyrolysis mass spectra (MS) as material descriptors. On the basis of these spectra, we construct prediction models using a data-driven approach, specifically focusing on thermal conductivity and adhesion, which are key indicators for heat dissipating performance. Pyrolysis-MS observes thermally decomposable polymers, which occupy only 0.1 volume fraction of the heat dissipators; nevertheless, the physical states of non-decomposable inorganic fillers are implicitly reflected in the pyrolyzed fragment patterns of the matrix polymers. Consequently, pyrolysis-MS provides sufficient information to construct accurate models for predicting heat dissipation performance, simplifying quality management by substituting time-consuming performance evaluations with rapid pyrolysis-MS measurements. Furthermore, we elucidate that higher crosslinking density of the matrix polymers enhances thermal conductivity. This data-driven method promises to streamline the identification of key functional factors in complex composite materials.

## IMPACT STATEMENT

Using pyrolysis-MS as a material descriptor allows for the prediction of composite materials’ heat dissipation capabilities and the identification of key factors influencing these properties.

## GRAPHICAL ABSTRACT

**Figure:**
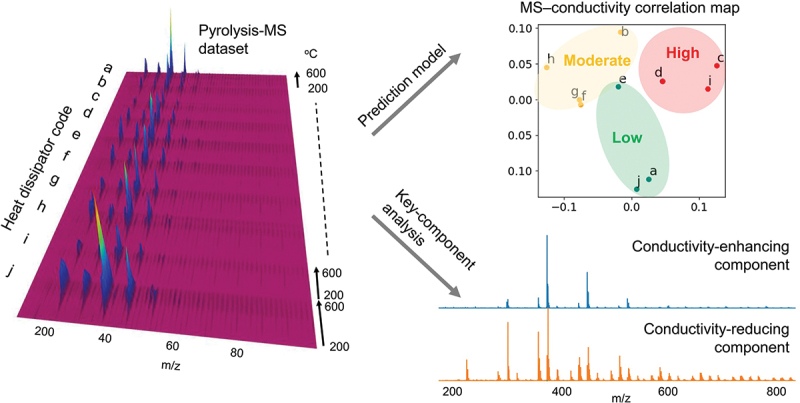

## Introduction

The dispersion and blending of inorganic fillers in a polymer matrix often improve their performance by magnitudes compared with pure polymeric materials [1]. This integration has notably transformed the field of heat dissipators, which are essential in various industrial applications. As electronic devices continue to improve in performance and become more compact, thermal management is growing in importance, particularly for enhancing the efficiency of heat conduction between two solid materials [2]. To this end, it is crucial not only to develop materials with high thermal conductivity in bulk but also to focus on the development of soft, adhesive materials that can efficiently fill the voids and maintain effective thermal contact at the interfaces of solid materials [2]. Composite heat dissipators emerge as an excellent choice for such materials, where the combination of inorganic fillers and matrix polymers enables the simultaneous achievement of high thermal conductivity and strong adhesion [3]. In particular, heat dissipators composed of inexpensive alumina fillers and silicone, known for its high heat resistance among polymers [4], are widely used in various industries and play an important role.

However, in composite materials, due to the complex interaction between inorganic fillers and matrix polymers, slight differences in structural parameters may cause significant variations in material properties [5]. Even if heat dissipators are the same according to the catalog, their heat dissipation characteristics can vary greatly from batch to batch. The structural parameters that potentially cause such variations include the particle size distribution and agglomeration of the filler, as well as the chemical structure and crosslinking density of the matrix polymer. However, since theoretical models are often oversimplified, it is challenging to clarify the structural parameters causing the variability [6]. Therefore, quality control process generally necessitates conducting time-consuming full inspections of many physical properties for each batch. Identifying the key factor for expressing heat dissipating properties on the basis of experimental data and a data-driven approach would facilitate quality control and also give insights toward material developments.

To address this challenge, we herein introduce pyrolysis mass spectra (MS) [7] as a material descriptor. Pyrolysis-MS measures pyrolyzed gaseous molecules generated from material samples heated up from room temperature to 600°C at a heating rate of 50°C/min. Inorganic fillers, which do not undergo thermal decomposition, cannot be directly observed by this measurement. Since inorganic fillers have a thermal conductivity higher than that of polymers by magnitudes, it is considered beneficial to add as much inorganic filler as possible while maintaining adhesion in heat dissipation agents [3,8]. The heat dissipators targeted in this study also consist of 90 wt% alumina filler and 10 wt% siloxane polymers. It seems challenging to infer the properties of the entire material from the information generated by very minor polymeric components. However, the physical state of the inorganic fillers may affect the thermal stabilities of polymers. Therefore, the fragment patterns of the pyrolyzed polymers may potentially embed information regarding the inorganic fillers, and detailed fragment pattern analysis through unsupervised machine learning may capture the physical properties of the entire heat dissipators. Therefore, we construct prediction models of thermal conductivity and adhesion on the basis of the MS-spectra – properties correlation analysis for 10 heat dissipators consisting of alumina and polydimethylsiloxane (PDMS). These models were applicable to silicone-based heat dissipators with different chemical structures, revealing that the minor polymer components contain information about the entire materials. Furthermore, a matrix polymer functions as a ‘bottleneck’ of thermal conduction [3,9,10]; therefore, improving the heat conductivity of the matrix polymer is crucial despite its small weight fraction [11]. In purely polymer-based heat dissipators without fillers, it is well known that higher crosslinking density leads to higher thermal conductivity [12,13]. The crosslinking density of a polymer network is usually calculated on the basis of viscoelastic measurements in accordance with the rubber elasticity theory [14]. However, applying such a method to composite materials consisting mostly of inorganic fillers makes it difficult to extract information about the polymer component, and to the best of our knowledge, few papers have discussed the crosslinking density of matrix polymer in composite materials. Our proposed method utilizing pyrolysis-MS inherently focuses on polymers rather than inorganic fillers, enabling accurate extraction of polymer structures. We experimentally clarify that heat dissipation agents with higher crosslinking density of the matrix polymer have higher thermal conductivities.

Accurate prediction models for material functions based on pyrolysis-MS would simplify the quality control process by replacing complex physical property evaluations with simple MS measurements. Furthermore, using the vast amount of data automatically collected in this process, important information for material development could be clarified. Thus, this paper is expected to provide a new perspective in understanding complex polymer-inorganic composite materials from the viewpoint of experiments and data science.

## Experimental section

### Preparation of heat dissipators

**Figure 1.**
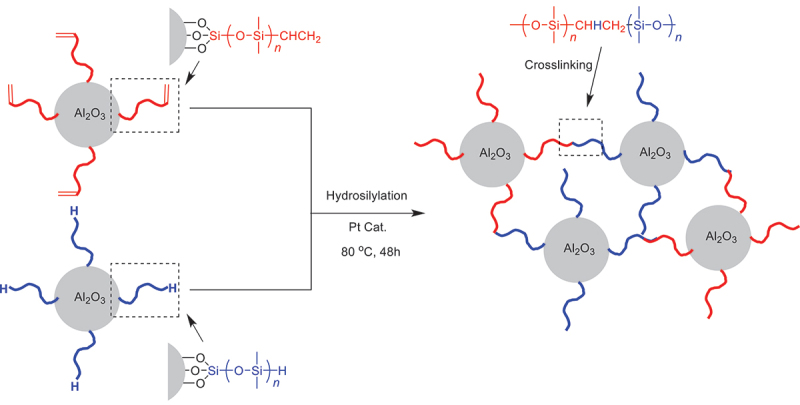
Synthetic scheme of alumina-silicone composite heat dissipators via hydrosilylation.

The process for preparing a typical heat dissipator is described as follows. Commercially-available alumina particles (particle size distribution: 10 ~ 200 um), modified with two types of PDMS, and a hydrosilylation platinum catalyst, were loaded into a mixing syringe in at a weight ratio of 1:1, along with 40 ppm (by weight) of a hydrosilylation platinum catalyst [15]. One type of alumina particles was modified with vinyl-terminated PDMS, while the other was modified with hydrogen-terminated PDMS, as illustrated in Figure 1. This mixture was kneaded using a twin-screw mechanism, extruded onto a glass plate, and then heated at 80°C for 48 hours to form crosslinked structures through hydrosilylation. Since the mono-functional reactive sites only existed on the polymer terminals, this ‘crosslinking’ reaction was a ‘chain-coupling’ reaction in a stringent definition; however, considering that each alumina particle possessed multiple polymer chains, the ‘chain-coupling’ reaction enabled the formation of a network structure. Therefore, although the reaction is technically a ‘chain-coupling’ reaction, its ability to create a network structure justifies referring to it as a ‘crosslinking’ reaction in this context. The resulted heat dissipators with network structures were then subjected to properties tests.

### Evaluation of heat dissipating performance

Heat conductivity measurements: Heat conductivities λ of dissipators were calculated on the basis of the following equation:

(1) $$\begin{array}{c} \lambda =C_{p}\rho \alpha , \end{array}$$

where $C_{p}$, ρ, and α are specific heat capacity, mass density, and thermal diffusivity, respectively, experimentally determined as follows.

The specific heat capacities were determined by differential scanning calorimetry (DSC) measurements (TA Instruments; USA). A heat dissipator specimen (20 mg) was placed in an aluminum pan and subjected to DSC measurement with a reference of sapphire. The specific heat capacity was calculated from the observed heat flow at a heating rate of 10°C/min from 0 to 150°C on the basis of the following equation:

(2) $$\begin{array}{c} C_{p}\left(\mathrm{sample}\right)=C_{p}\left(\mathrm{sapphire}\right)\frac{H\left(\mathrm{sample}\right)-H\left(\mathrm{empty}\right)}{H\left(\mathrm{sapphire}\right)-H\left(\mathrm{empty}\right)}\frac{m\left(\mathrm{sapphire}\right)}{m\left(\mathrm{sample}\right)}, \end{array}$$

where *H* and *m* are heat flow and mass, respectively.

The mass densities were determined on the basis of the Archimedes method. The weight of a dissipator film of 1 cm^2^ with a 1-mm thickness were measured and correlated to its volume precisely determined using a hydrometer (ALFA MIRAGE; Japan, water was used as a density-known liquid).

The thermal diffusivity was measured by temperature wave thermal analysis (TWTA; ai-PHASE; Japan). Temperature waves at a frequency of 0.02 ~ 100 Hz were applied to a dissipator film of 1 cm^2^ with a 1-mm thickness at room temperature, and thermal diffusivity was calculated from the phase delay on the basis of the following equation:

(3) $$\begin{array}{c} \alpha =\frac{\pi fd^{2}}{{\left(\Delta \theta +\frac{\pi }{4}\right)}^{2}} \end{array}$$

where *f*, *d*, and Δθ are frequency, thickness, and phase difference, respectively.

Adhesion measurements: Adhesion was determined by a 90°-peel test against a thin aluminum sheet attached to the heat dissipators’ surface. Heat dissipators were attached to the thin aluminum sheet with a diameter of 100 mm, and cured at room temperature for at least 3 days. After curing, the peeling strength was measured using the force gauge (Imada; Japan) with a peel rate of 50 mm/min.

### Measurements of pyrolysis-MS

**Figure 2.**
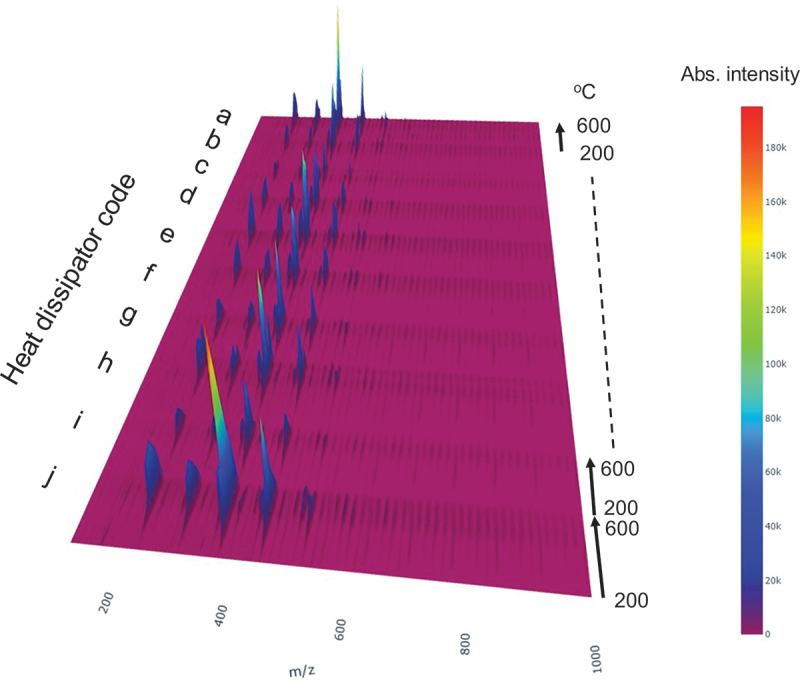
Pyrolysis-MS 2D spectra of the training dataset, including heat dissipators labeled a–j, were analyzed. All peaks originated from PDMS, resulting in consistent peak positions along the m/z axis within this dataset. The primary peak series corresponded with cyclic dimethylsiloxane oligomers, varying in ring member numbers. However, the intensity ratio of each peak within a spectrum differed, reflecting the diverse thermal conditions of the polymers influenced by individually different crosslinking densities and structural parameters of the alumina fillers.

Each heat dissipator sample, weighing 1.0 mg, underwent a pyrolysis process. This was conducted using an ionRocket heater (BioChromato; Japan), where the sample was heated at a rate of 50°C/min, starting from an initial temperature of 50°C, which was maintained for a preheating duration of two minutes, reaching up to 600°C. The entire process for each sample spanned 13 minutes. Too rapid heating rate may create a temperature distribution within the sample, potentially distorting the temperature profile. On the other hand, an excessively slow heating rate should also be avoided for efficient dataset creation. As a preliminary study, we measured the pyrolysis-MS of the dissipator d at varying heating rates of 25, 50, and 100°C/min. While the temperature profiles at 25°C/min and 50°C/min were almost identical, the profiles at 50°C/min and 100°C/min were completely different (Fig. S1). Therefore, we adopted 50°C/min as the optimal heating rate. During pyrolysis, the evolved gases were ionized using a DART-ion source (DART-OS ionSense; Bruker; USA) with excited helium gas. The resulting mass spectra were collected using an LCMS-2020 (Shimadzu; Japan) in the positive-ion mode, capturing data at a frequency of 50 scans per minute, resulting in a total of 650 spectra for each sample. The spectra covered a mass-to-charge ratio (m/z) range from 100 to 1000 m/z, with a 0.05-m/z interval scale and a mass resolution of 2000. Finally, the spectral data were exported in the CDF file format and subsequently converted into the Numpy 3.9 format using the netCDF4 Python module. The spectra were further formatted for data size reduction; spectra in the temperature range of 200–600°C, which is critical for polymer thermal decomposition, were extracted and divided into eight segments, each corresponding to a 50°C increment. The formatted spectral dataset was visualized as a 3D plot (*m/z–*temperature – intensity) shown in Figure 2, of which numerical data can be found in Data S1.

## Results and discussions

**Figure 3.**
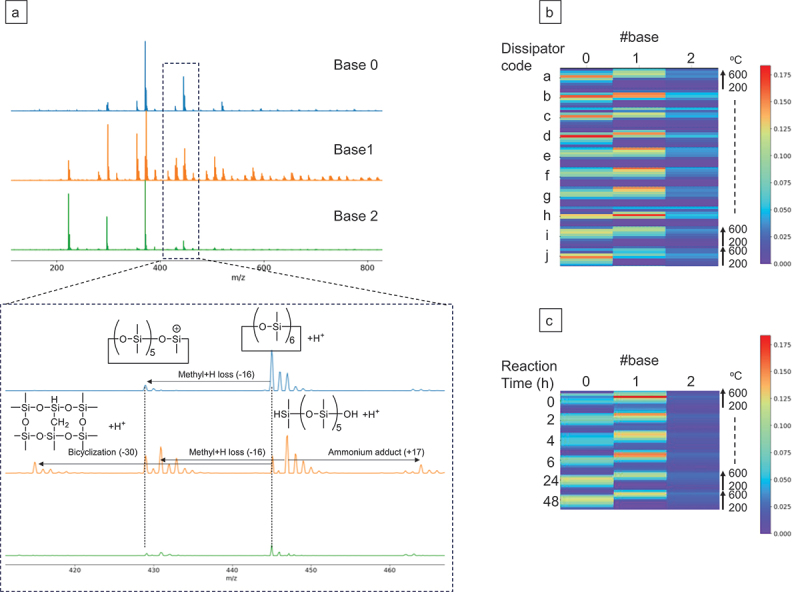
Extracted basis spectra (a), their abundances across the training dataset (b), and reaction-time variable dataset (c). The inset shows the magnified spectra around 440 m/z. The basis number of three was automatically determined by ARD.

Pyrolysis-MS is expected to contain not only information about thermally-decomposable polymers but also essential information about the entire composite materials. However, pyrolysis-MS is a complex two-dimensional spectrum that includes not only a *m/z* axis but also a decomposition temperature axis, making it difficult for scientists to extract important information [16]. Figure 2 shows the spectral dataset of 10 commercially available heat dissipators provided by different suppliers, all consisting of 90 wt% alumina fillers and 10 wt% PDMS. Although these heat dissipators were chemically equivalent according to catalogs, they exhibited markedly different pyrolysis-MS spectra (Figure 2). Each sample had 650 spectra recorded at temperatures ranging from 200 to 600°C (heating rate: 50°C/min), which were divided and averaged into eight spectra per sample. The dataset thus consisted of 80 spectra (all spectra are presented in Data S1). To intuitively understand the spectral variations across the dataset and simplify the spectra – properties correlation analysis, we first decomposed the complex raw spectra into base spectra of pyrolysis fragments. Non-negative matrix factorization (NMF) [17,18], an unsupervised learning technique long studied in the machine learning community, was applicable to this purpose (see our implementation of NMF to pyrolysis-MS analysis in the previous report [16]. Figure 3a–b shows the three base spectra extracted by NMF and their abundances across the dataset. Note that the number of the base spectra was automatically determined on the basis of sparse modeling assumption well known as the automatic relevance determination (ARD) method [18].

Our first attempt was to comprehend the rationale behind the AI’s generation of these three spectra for summarizing the entire spectral dataset, as well as the significance of this decision. According to the synthetic scheme (Figure 1), the polymer species that could potentially be observed in pyrolysis-MS were uncross-linked PDMS with H- or vinyl-terminals and those crosslinked through hydrosilylation [15]. The most straightforward interpretation was that these three base spectra reflected differences in such terminal structures of three kinds of polymers. However, unlike matrix-assisted laser desorption/ionization time-of-fly MS (MALDI-TOF-MS), which can directly observe individual polymer chains without fragmentations but is generally limited to linear soluble polymers with low molecular weight (*m/z* < 20,000), pyrolysis-MS observes volatile short fragments (*m/z <* 1000) thermally desorbed from the main chain. Therefore, in high molecular-weight polymers over 100,000 Da, fragments with end-terminal structures would be almost undetectable as compared with fragments from inner main chains. To clarify this, two kinds of PDMS with H- and vinyl-terminals were measured individually by pyrolysis-MS (Fig. S2). They looked almost identical, demonstrating that it is impossible to distinguish terminal structures of high molecular-weight polymers using pyrolysis-MS.

We then hypothesized that the thermal stabilities of polymer chains changed depending on the degree of crosslinking, affecting the fragmentation modes during thermal decomposition, and these differences were extracted as the distinct base spectra. To validate this hypothesis, we prepared six samples with different crosslinking densities by varying the reaction time of hydrosilylation (reaction times: 0, 2, 4, 6, 24, and 48 hours at 30°C). Projecting the observed spectra of these samples onto the subspace spanned by the aforementioned three base spectra revealed that samples with higher crosslinking densities, i.e. samples with longer reaction times, had more of base 0 and less of base 1 (see Figure 3c; also refer to Fig. S3 for an easier comparison of the difference in basis abundances). Thus, it was concluded that base 0 was derived from crosslinked structures, while base 1 was from uncross-linked siloxanes. Referring to the previously reported peak attributions in pyrolysis-MS of PDMS [19–21], the chemical structural formulas deduced from each m/z value are presented in Figure 3a. Both base 0 and 1 originate from the PDMS main chain; however, the peak pattern of base 1 (non-linking structure) was considerably more complex compared with base 0 (linking structure). This difference is mainly attributable to the difference in the temperature range at which the fragments were generated; according to Figure 3b-c, fragments of base 1 were primarily generated at temperatures about 50 to 100°C higher than those of base 0. As a result, various reactions such as methyl translation and bicyclization further progressed, leading to the formation of relatively unstable gas molecules with Si-H bonds. Overall, although pyrolysis-MS cannot directly observe the terminal fragment changes derived by crosslinking reactions in high-molecular-weight polymers, spectral unsupervised learning visualized the difference in the crosslinking densities by successfully extracting the decrease in thermal stability caused by crosslinking. A data-driven approach that extracts common peak patterns across the dataset can readily visualize such complex physical transformation phenomena. While base 0 and 1 demonstrated clear increasing and decreasing trends, respectively, due to the variable reaction times, base 2 did not show a significant difference. Therefore, base 2 was likely attributable to fragments originating from domains where thermal properties did not significantly vary by crosslinking. Such domains could be situated near the end fixed onto alumina filler.

**Figure 4.**
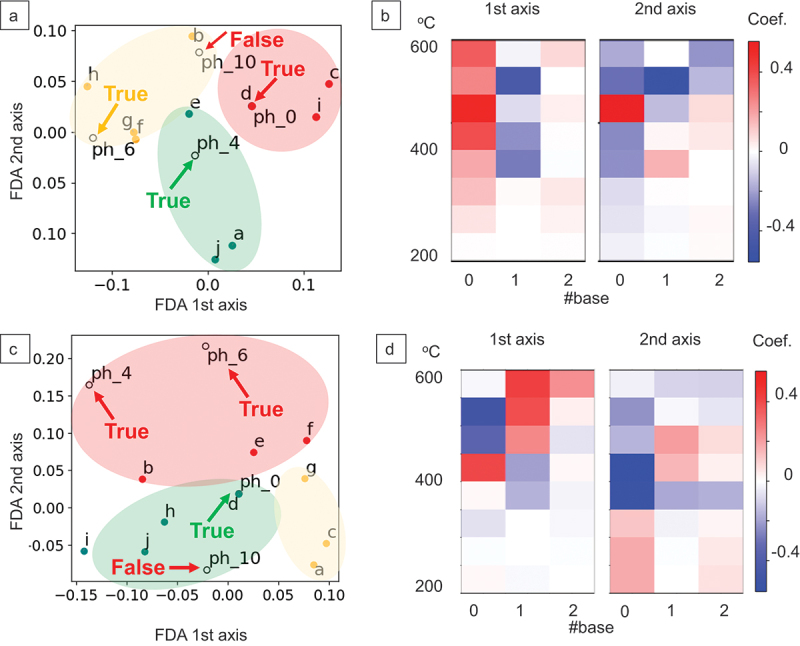
FDA for spectra-heat conductivity (a-b) and spectra-adhesion correlation analysis (c-d). The red, yellow and green regions represent high, moderate and low thermal conductivity (a) and adhesion (b), respectively. The predicted classifications of test samples were marked as ‘True’ or ‘False’ by comparing answers in Table 1. (b, d) illustrates the coefficients of the linear combination in the 24-dimensional feature vector space as matrices with a size of (8, 3) for synthesizing the FDA axes.

Next, we attempted to construct prediction models for material properties on the basis of the abundances of these three base spectra as a descriptor. Note that the descriptor retained the abundances of the base spectra for each of the decomposition temperature ranges divided into eight. Therefore, each heat dissipator had a 24-dimensional feature vector. Since the size of the dataset was much smaller than the dimensional number of the feature vectors, dimension reduction using principal component analysis (PCA) was conducted so that 99% of the variance was retained, reducing the dimensional number down to eight. Subsequently, a Fisher discriminant analysis (FDA) [22] was conducted to analyze spectra – properties correlations. FDA is a classification method based on subspace learning, which minimizes the variance *within* classes and maximizes the variance *between* classes over the training dataset. Feature vectors can be then projected onto the leaned low-dimensional space. First, we labeled the training data of 10 alumina-PDMS heat dissipators into three classes – high, moderate, and low – on the basis of their numerical data of thermal conductivity and adhesion (Table 1). The FDA based on this classification is shown in Figure 4. Note that tight clustering of training data does not necessarily mean the learned subspace is useful; the key question is whether the test data can be correctly classified when projected onto this learned subspace. In addition, the correct classification of test data is simpler when it resembles some training data, compared with when it significantly differs from any training data. In particular, in data-driven materials research with extremely small datasets, like in this study, it is crucial to define the applicable material space for the learned prediction model. Therefore, we tested heat dissipators with slight differences in polymer chemical structures from the alumina-PDMS composites of the training data to assess the extent to which the prediction model can be applied to chemically altered structures. To this end, we used heat dissipators containing poly(dimethylsiloxane-co-methylphenylsiloxane), where {0, 4, 6, 10} mol% of the methyl groups in PDMS were replaced with phenyl groups, as test data (alumina filler content was consistently 90 wt%). As shown in Figure 4a and c, the prediction models for thermal conductivity and adhesion correctly classified the test samples with 0, 4, 6 mol% phenyl substitution, but failed for 10 mol% substitutions. The robustness of this model, which was trained only on datasets of homopolymers, is surprising, as the pyrolysis-MS spectra of random copolymers are known to shift significantly along the m/z axis even when the minor monomer content is below 10 mol%. This robustness could be attributable to the unchanged state of the alumina fillers captured through the polymers. Expanding the dataset would lead to prediction models covering a broader material space. Moreover, FDA projection axes can provide important insights. Figure 4b shows the two axes defining the leaned FDA subspace in the prediction model of thermal conductivity, represented as a matrix whose size was (temperature band number, base number) = (8, 3). The dimensions marked in red (or blue) indicated that feature vectors containing more (or less) of the corresponding base at the corresponding temperature were plotted in the positive direction along that axis. Figure 4a shows that samples distributed in the positive direction of both primary and secondary axes demonstrated high thermal conductivity. Considering how the primary axis was synthesized from Figure 4b, it was clear that samples with more of base 0 and less of base 1 across all temperature ranges were projected in the positive direction (rightward) on the primary axis in Figure 4a. Therefore, we concluded that a higher quantity of base 0 fragments, derived from crosslinked structures, and a lower quantity of base 1 fragments, derived from uncross-linked structures, indicate high thermal conductivity. This conclusion was derived purely from numerical correlation analysis, and it is unclear whether these correlations imply causality. A common issue in materials informatics relevant to the small dataset size is the potential observation of spurious correlations by chance. While scrutiny of the validity of these results is necessary, it is well-known in systems without fillers that higher cross-link density correlates with increased thermal conductivity, lending credence to the conclusion as being reasonable. As for the secondary axis, although it is difficult to discuss since the sign was reversed depending on the temperature for the same base, it complemented temperature distribution information not well-captured by the primary axis, to further refine thermal conductivity predictions by considering both the amount and the thermal environments of crosslinking structures. The clear and interpretable correlation between thermal conductivity and pyrolysis-MS arose because the pyrolysis fragmentation patterns reflect differentiated cross-link densities in matrix polymers, which also impact thermal conductivity. It is important to compare our data-driven prediction model with previously reported theoretical models [23] for heat conductivity in composite dissipators. We utilized the thermal conductivities of PDMS at 0.2 Wm^−1^K^−1^ and alumina at 29 Wm^−1^K^−1^, with alumina’s weight fraction set at 0.9, and performed thermal conductivity calculations using the series model, parallel model, and co-continuous model, obtaining results of 0.65 Wm^−1^K^−1^, 20 Wm^−1^K^−1^, and 4.8 Wm^−1^K^−1^, respectively. The co-continuous model most effectively accounted for the experimental values, which ranged from 1.1 to 5.2 Wm^−1^K^−1^. However, considering that the alumina weight fraction remained constant across all 14 heat sinks, and with the experimental thermal conductivity ranging from 1.1 to 5.2 Wm^−1^K^−1^, it appears necessary to hypothesize the presence of some isolated dispersed phases instead of a completely co-continuous phase when constructing the physical model. Measuring such a degree of dispersion experimentally poses significant challenges. Additionally, it is crucial to account for variations in the thermal conductivity of the polymer phase due to its molecular structure and geometry, as well as to consider the interface thermal resistance. While physical models are instrumental in understanding phenomena, accurately calculating the desired properties requires experimental determination of various unknown parameters, and the discrepancy between calculated values and actual measurements tends to widen with the complexity of the phenomena addressed. Conversely, our proposed data-driven model can predict the desired properties solely based on the correlations from MS spectra, thereby reducing the impact of system complexity and providing a significant advantage in conveniently achieving objectives.

**Table 1.** Properties of heat dissipators used in this work.

Code^[a]^	Provider Catalog No.	Phenyl^[b]^ fraction [mol%]	Heat conductivity [Wm^−1^K^−1^]	Adhesion [Jm^−2^]	Heat^[c]^ conductivity label	Adhesion^[d]^ label
a	Shinetsu SDP1030	0	1.1	51	L	M
b	Shinetsu SDP2060	0	2.1	139	M	H
c	Shinestu SDP3540	0	3.1	38	H	M
d	Shinestu SDP5040	0	5.2	30	H	L
e	Dow Toray TC4515	0	1.3	479	L	H
f	Dow Toray TC4525	0	2.0	94	M	H
g	Dow Toray TC4535	0	2.9	36	M	M
h	Denka GFCR1	0	2.0	25	M	L
i	Denka GFCR8	0	3.2	15	H	L
j	Denka GFCNH1	0	1.3	31	L	L
ph_0	-	0	5.1	28	-	-
ph_4	-	4	1.6	166	-	-
ph_6	-	6	2.0	160	-	-
ph_10	-	10	4.0	140	-	-

[a] The training dataset consisted of a to j, while test dataset consisted of ph_0 to ph_10.

[b] The percentage of methyl groups in PDMS replaced by phenyl groups.

[c] Heat dissipators were labeled on the basis of their thermal conductivity [Wm-1K–1]: those with a value less than 2 were labeled as L, those between 2 and 3 as M, and those greater than 3 as H. The test data were not labeled but were inferred by the prediction model.

[d] Heat dissipators were labeled on the basis of their adhesive [Jm-2]: those with a value less than 35 were classified as L, those between 35 and 70 as M, and those greater than 70 as H. The test data were not labeled manually but were inferred by the prediction model.

While the prediction process of thermal conductivity was interpretable as mentioned above (Figure 4b), that of adhesion was less interpretable (Figure 4d). Unlike thermal conductivity, adhesion was not directly influenced by the quantity of crosslinking structures but appeared to be indirectly affected by the thermodynamic stability of both crosslinked and linear structures. For instance, by combining Figure 4C-D, it was observed that adhesion increased when fragment 0, originating from crosslinked structures, was abundant at low temperatures, and decreased when it is abundant at high temperatures. This suggests a correlation between the environment surrounding the crosslinked structures and adhesion, regardless of whether there is a causal relationship. While a more sophisticated physical model is necessary to fully understand this mechanism, accurate predictive models for adhesion can still be constructed based on these correlations, as shown in Figure 4c. This demonstrates that pyrolysis-MS measurements can effectively replace thermal conductivity and adhesion tests.

## Conclusion

This study has demonstrated that the properties of polymer-inorganic composite heat dissipators can be estimated from pyrolysis-MS. Although it cannot directly observe non-thermally decomposable inorganic materials, the thermal decomposition patterns of polymers, which occupied only 10 wt% of the entire materials, implicitly embed information about the thermal environment constructed by the surrounding inorganic fillers. By correlating spectral descriptors with material properties, we elucidated that it is possible to predict material properties and identify their key factors efficiently. In composite materials with minimal polymer content, analytically focusing on the polymer has been challenging. Pyrolysis-MS would be a key analytical tool, especially when the minor polymer part is the ‘bottleneck’ in the performance expression. We elucidated for the first time that the crosslinking density of the matrix polymer in composites was a governing factor in thermal conductivity. We also demonstrated simultaneous prediction of seemingly unrelated properties such as thermal conductivity and adhesion on the basis of a single spectral descriptor. Therefore, pyrolysis-MS can potentially replace and consolidate numerous inspection items in quality control processes. The data obtained as a byproduct in this process augments the dataset, which would broaden the predictable material space and also elucidate guidelines to material development.

## Supplementary Material

Supplemental Material

## Data Availability

Spectral dataset including all the spectra used in this study is attached as Data S1.
